# Candidalysins Are a New Family of Cytolytic Fungal Peptide Toxins

**DOI:** 10.1128/mbio.03510-21

**Published:** 2022-01-25

**Authors:** Jonathan P. Richardson, Rhys Brown, Nessim Kichik, Sejeong Lee, Emily Priest, Selene Mogavero, Corinne Maufrais, Don N. Wickramasinghe, Antzela Tsavou, Natalia K. Kotowicz, Olivia W. Hepworth, Ana Gallego-Cortés, Nicole O. Ponde, Jemima Ho, David L. Moyes, Duncan Wilson, Christophe D’Enfert, Bernhard Hube, Julian R. Naglik

**Affiliations:** a Centre for Host-Microbiome Interactions, Faculty of Dentistry, Oral and Craniofacial Sciences, King’s College London, London, United Kingdom; b Department of Microbial Pathogenicity Mechanisms, Leibniz Institute for Natural Product Research and Infection Biology, Hans Knoell Institute, Jena, Germany; c Institut Pasteur, Université de Paris, Bioinformatics and Biostatistics Hub, Paris, France; d Institut Pasteur, Université de Paris, INRAE, USC2019, Unité Biologie et Pathogénicité Fongiques, Paris, France; e Medical Research Council Centre for Medical Mycology at the University of Exeter, Exeter, United Kingdom; f Institute of Microbiology, Friedrich Schiller University, Jena, Germany; Geisel School of Medicine at Dartmouth

**Keywords:** *Candida*, candidalysin, fungus, peptide toxin

## Abstract

Candidalysin is the first cytolytic peptide toxin identified in any human fungal pathogen. Candidalysin is secreted by Candida albicans and is critical for driving infection and host immune responses in several model systems. However, *Candida* infections are also caused by non-C. albicans species. Here, we identify and characterize orthologs of C. albicans candidalysin in C. dubliniensis and C. tropicalis. The candidalysins have different amino acid sequences, are amphipathic, and adopt a predominantly α-helical secondary structure in solution. Comparative functional analysis demonstrates that each candidalysin causes epithelial damage and calcium influx and activates intracellular signaling pathways and cytokine secretion. Importantly, C. dubliniensis and C. tropicalis candidalysins have higher damaging and activation potential than C. albicans candidalysin and exhibit more rapid membrane binding and disruption, although both fungal species cause less damage to epithelial cells than C. albicans. This study identifies the first family of peptide cytolysins in human-pathogenic fungi.

## INTRODUCTION

Cytolytic proteins and peptide toxins are classical virulence factors of bacterial pathogens and play a major role in bacterial disease (1, 2). Human-pathogenic fungi were not known to possess such toxins until the recent discovery of candidalysin, a peptide toxin secreted by Candida albicans (3). C. albicans is normally a benign member of the microbiota (4), but under certain predisposing conditions, it can cause superficial mucosal infection in healthy individuals and potentially fatal invasive and systemic infection in the immunocompromised (5).

Candidalysin is critical for C. albicans mucosal infections and is produced by targeted proteolytic processing of the hypha-associated protein Ece1p by kexin proteases at conserved lysine-arginine recognition sites (6, 7). Following secretion, candidalysin destabilizes the plasma membrane of epithelial cells (3, 8), triggers cellular stress resulting in necrotic cell death (9), and facilitates fungal translocation across gastrointestinal epithelial cells (10).

Host recognition of candidalysin activity comprises a critical aspect of immune defense against C. albicans. Candidalysin activates the epidermal growth factor receptor (EGFR) (11) and triggers innate epithelial immune responses predominantly through the mitogen-activated protein kinase (MAPK) pathway and the activating protein 1 (AP-1) transcription factor c-Fos that drives numerous cytokine responses, a process that is regulated by the MAPK phosphatase MKP1 (via the extracellular signal-regulated kinase 1 and 2 [ERK1/2] MAPK pathway) (3, 12, 13). Epithelial recognition of candidalysin activity subsequently drives a number of critically important innate cellular immune responses, including the proliferation of tissue-resident interleukin-17 (IL-17)-producing CD4^+^ T cell receptor αβ-positive (TCRαβ^+^) type 17 immune cells in murine oral tissue (14), neutrophil recruitment to the kidney (15) and brain (16) during murine systemic infection, and the secretion of IL-1β from macrophages via NLRP3 inflammasome activation (17, 18). Candidalysin also elicits protective allergic responses via platelet-mediated T helper 2 (Th2) and Th17 cell polarization (19).

Although C. albicans is associated with the majority of mucosal *Candida* infections, candidiasis is also caused by several non-C. albicans species, including C. dubliniensis and C. tropicalis, which induce varied epithelial responses *in vitro* and *in vivo* (20–24). While C. albicans, C. dubliniensis, and C. tropicalis exhibit differences in their abilities to maintain hyphae on epithelial cells, all three species cause epithelial damage (although to different extents) and stimulate the release of the damage-associated cytokine IL-1α (23, 25–27). Orthologs of C. albicans *ECE1* have been identified in C. dubliniensis and C. tropicalis, which exhibit differences in gene expression *in vitro* and *in vivo* in the context of vaginal immunopathology (24). However, it is unknown whether the *ECE1* genes of C. dubliniensis and C. tropicalis encode candidalysin toxins and whether these are biologically functional.

Here, we characterize the biological function of the candidalysin toxins from C. dubliniensis and C. tropicalis. Investigations with oral epithelial cells and artificial lipid membranes demonstrate that the C. dubliniensis and C. tropicalis candidalysins possess more potent cytolytic and immunostimulatory activity than C. albicans candidalysin despite the paradox that C. dubliniensis and C. tropicalis are considered less “pathogenic” than C. albicans.

This work identifies the candidalysins as a novel family of fungal peptide toxins that are functionally conserved but differ in potency.

## RESULTS

### Identification of candidalysin toxins in C. dubliniensis and C. tropicalis.

**FIG 1:**
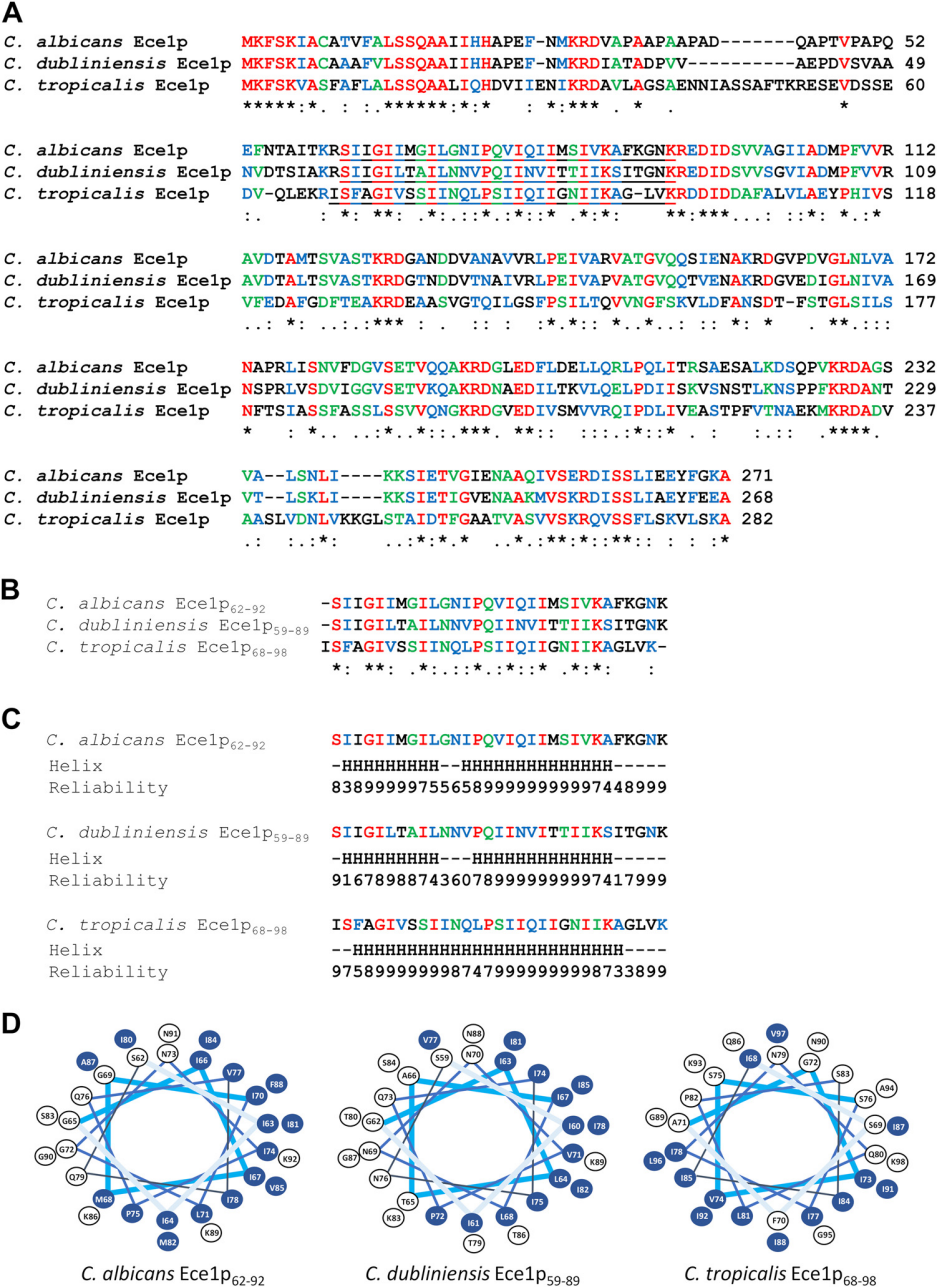
Identification of a candidalysin toxin in Candida dubliniensis and Candida tropicalis. (A) Clustal Omega multiple-sequence alignment of Ece1p from C. albicans, C. dubliniensis, and C. tropicalis. Asterisk (*), conserved residues (red); colon (:), residues with strongly similar properties (blue); period (.), residues with weakly similar properties (green). Regions corresponding to candidalysin-like peptides are underlined. (B) Clustal Omega multiple-sequence alignment of candidalysin peptides from C. albicans, C. dubliniensis, and C. tropicalis. (C) JPred secondary structure analysis of candidalysin peptides from C. albicans, C. dubliniensis, and C. tropicalis. Regions predicted to form an α-helix are identified (“H”). The reliability of prediction is scored numerically (0 to 9), where 0 is the weakest and 9 is the strongest. (D) Helical-wheel renderings of candidalysin sequences from C. albicans, C. dubliniensis, and C. tropicalis. Hydrophobic amino acid residues are highlighted in blue.

To identify candidalysin toxins in pathogenic non-C. albicans species of *Candida*, we used the amino acid sequence of C. albicans SC5314 Ece1p to screen the PhylomeDB v4 database (28) and performed a BLASTp search at the National Center for Biotechnology Information (NCBI) for Ece1p orthologs. The screen identified Ece1p orthologs in the closely related fungal pathogens C. dubliniensis and C. tropicalis. Clustal Omega multiple-sequence alignment (29) of C. albicans Ece1p and the Ece1p sequences of C. dubliniensis and C. tropicalis (Fig. 1A) revealed the presence of a candidalysin-like region (designated C. dubliniensis Ece1p_59–89_ and C. tropicalis Ece1p_68–98_) flanked by lysine-arginine kexin recognition sites, similar to C. albicans candidalysin (Ece1p_62–92_) (Fig. 1B). Analysis of each candidalysin-like sequence using the JPred 4 protein secondary structure prediction server (30) indicated the presence of two distinct α-helix-forming regions in the peptides of C. albicans and C. dubliniensis but only a single α-helix-forming region in the peptide of C. tropicalis (Fig. 1C).

Helical-wheel renderings (31) of C. dubliniensis Ece1p_59–89_ and C. tropicalis Ece1p_68–98_ revealed an asymmetric distribution of hydrophobic amino acids similar to that of C. albicans candidalysin (Ece1p_62–92_) (Fig. 1D). Analysis of each sequence using the TANGO algorithm (32) suggested that the peptides of C. albicans and C. dubliniensis, but not that of C. tropicalis, were similar in their predicted abilities to form helical aggregates (see Fig. S1A in the supplemental material). Together, these data indicate that candidalysin-like toxin sequences exist in C. dubliniensis and C. tropicalis (termed candidalysins herein).

10.1128/mbio.03510-21.1 FIG S1 (A) Percent helix aggregation of candidalysins from C. albicans, C. dubliniensis, and C. tropicalis. Analysis of candidalysin toxins from C. albicans, C. dubliniensis, and C. tropicalis was performed by using the TANGO algorithm. Regions predicted to undergo helical aggregation within each candidalysin are indicated. (B) Quantification of calcium influx in TR146 oral epithelial cells treated with candidalysins. Calcium influx in epithelial cells treated with the candidalysins (70 μM) of C. albicans, C. dubliniensis, and C. tropicalis was determined. Statistics are applied relative to vehicle-treated cells. Data are presented as means and standard deviations (SD) from 3 biological repeats. Statistical significance was calculated using one-way ANOVA with Tukey’s *post hoc* comparison test. ****, *P ≤ *0.0001. (C) Candidalysins exhibit different binding affinities for planar 1,2-diphytanoyl-*sn*-glycero-3-phosphocholine (DPhPC) lipid bilayers. Candidalysins from C. albicans, C. dubliniensis, and C. tropicalis (2, 4, 6, 8, and 10 μM) were applied to DPhPC planar lipid bilayers, and the mean dwell time (defined as the time that elapsed between candidalysin addition and bilayer permeabilization) was quantified. Data are presented as means and SD from 10 biological repeats. Download FIG S1, TIF file, 0.2 MB. Copyright © 2022 Richardson et al. 2022 Richardson et al. https://creativecommons.org/licenses/by/4.0/ This content is distributed under the terms of the Creative Commons Attribution 4.0 International license.

### C. dubliniensis and C. tropicalis candidalysins are α-helical cytolysins that exhibit potent damage-inducing ability.

**FIG 2:**
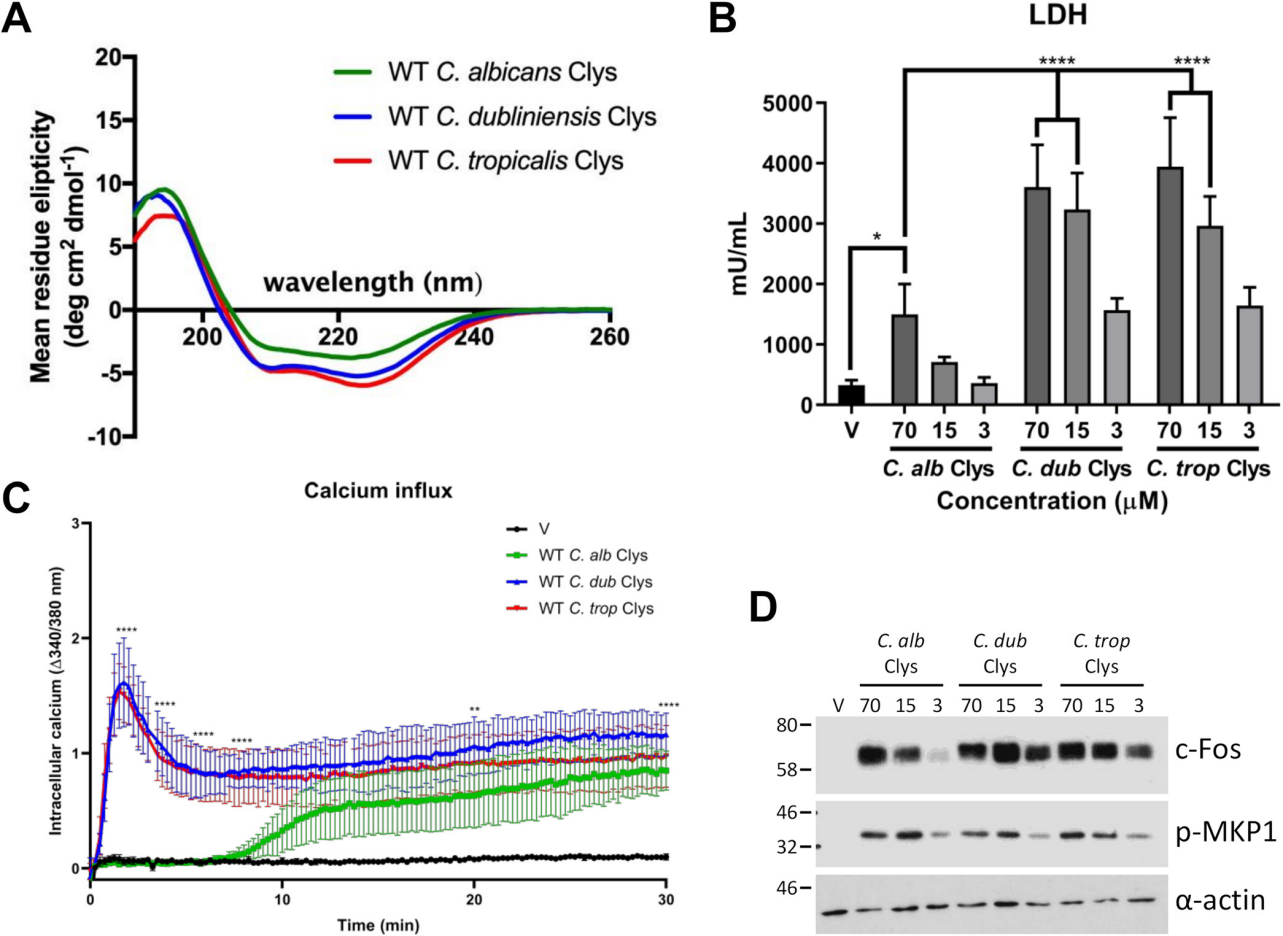
The candidalysin family of cytolysins exhibits different potencies on TR146 oral epithelial cells. (A) Circular dichroism spectroscopy of candidalysin (Clys) toxins from C. albicans, C. dubliniensis, and C. tropicalis. (B) TR146 oral epithelial cells were treated with candidalysins (70, 15, and 3 μM) from C. albicans, C. dubliniensis, and C. tropicalis for 24 h, and levels of cell damage were assessed by an LDH assay. Statistics are applied relative to 70 μM C. albicans candidalysin. For C. albicans candidalysin (3 μM) versus C. dubliniensis (3 μM) and C. tropicalis (3 μM) candidalysins, the *P* values were 0.0016 (**) and 0.0006 (***), respectively (*n* = 6 biological repeats). (C) Calcium influx in epithelial cells treated with the candidalysins (70 μM) of C. albicans, C. dubliniensis, and C. tropicalis for 30 min. Statistics are applied relative to WT C. albicans candidalysin (2, 4, 6, and 8 min) and vehicle-treated (V) cells (20 and 30 min) (*n* = 3 biological repeats). (D) Western blot analysis of epithelial cells treated with the candidalysins (70, 15, and 3 μM) of C. albicans, C. dubliniensis, and C. tropicalis for 2 h. Epithelial cell lysates (5 μg total protein) were probed with anti-c-Fos and anti-p-MKP1 antibodies. One representative blot is presented (from 3 biological repeats). Statistics are applied relative to vehicle-treated cells (*n* = 3 biological repeats). For panels B and C, data are presented as means and standard deviations (SD). Statistical significance was calculated using one-way ANOVA with Tukey’s *post hoc* comparison test. ****, *P ≤ *0.0001; **, *P ≤ *0.01; *, *P ≤ *0.05.

To investigate the biological activity of these newly discovered candidalysins, peptides corresponding to C. dubliniensis Ece1p_59–89_ (SIIGILTAILNNVPQIINVITTIIKSITGNK) and C. tropicalis Ece1p_68–98_ (ISFAGIVSSIINQLPSIIQIIGNIIKAGLVK) were synthesized, and their biophysical and biological properties were compared to those of C. albicans candidalysin (SIIGIIMGILGNIPQVIQIIMSIVKAFKGNK). Circular dichroism (CD) spectroscopy analysis revealed that the C. dubliniensis and C. tropicalis candidalysins adopt a predominantly α-helical secondary structure in solution, similarly to C. albicans candidalysin (Fig. 2A).

C. albicans candidalysin causes epithelial damage, which can be measured by the release of lactate dehydrogenase (LDH) (3, 8, 10). Therefore, we assessed the ability of each newly discovered candidalysin to cause epithelial damage by treating TR146 oral epithelial cells with the three candidalysins (70, 15, and 3 μM, representing highly lytic, moderately lytic, and sublytic concentrations of C. albicans candidalysin) for 24 h and quantified LDH activity. As expected, the C. albicans candidalysin caused significant damage in a dose-dependent manner. Surprisingly, while C. dubliniensis and C. tropicalis fungi cause less damage to epithelial cells than C. albicans (23), the candidalysins of C. dubliniensis and C. tropicalis caused significantly greater damage at all concentrations than C. albicans candidalysin (Fig. 2B).

C. albicans candidalysin causes calcium influx into epithelial cells (3). Accordingly, we investigated the ability of C. dubliniensis and C. tropicalis candidalysins to induce calcium influx in oral epithelial cells. All three candidalysins induced a significant level of calcium influx between 30 and 180 min (Fig. S1B). However, C. dubliniensis and C. tropicalis candidalysins induced significantly more rapid calcium influx than C. albicans candidalysin within the first 10 min (Fig. 2C). These data demonstrate that the C. dubliniensis and C. tropicalis candidalysins are α-helical peptide toxins that are more potent in their ability to cause epithelial damage and calcium influx than C. albicans candidalysin.

### C. dubliniensis and C. tropicalis candidalysins potently activate epithelial immune responses.

C. albicans candidalysin activates epithelial cells via the MAPK pathway comprising c-Fos and MKP1 signaling (3, 8, 12, 13, 33). To determine whether the C. dubliniensis and C. tropicalis candidalysins induce similar responses, oral epithelial cells were treated with all three candidalysins (70, 15, and 3 μM) for 2 h, and c-Fos expression/MKP1 phosphorylation was assessed by Western blotting. All candidalysins activated epithelial c-Fos/p-MKP1 signaling in a dose-dependent manner; however, C. dubliniensis and C. tropicalis candidalysins upregulated c-Fos more potently at lower concentrations than C. albicans candidalysin (Fig. 2D).

**FIG 3:**
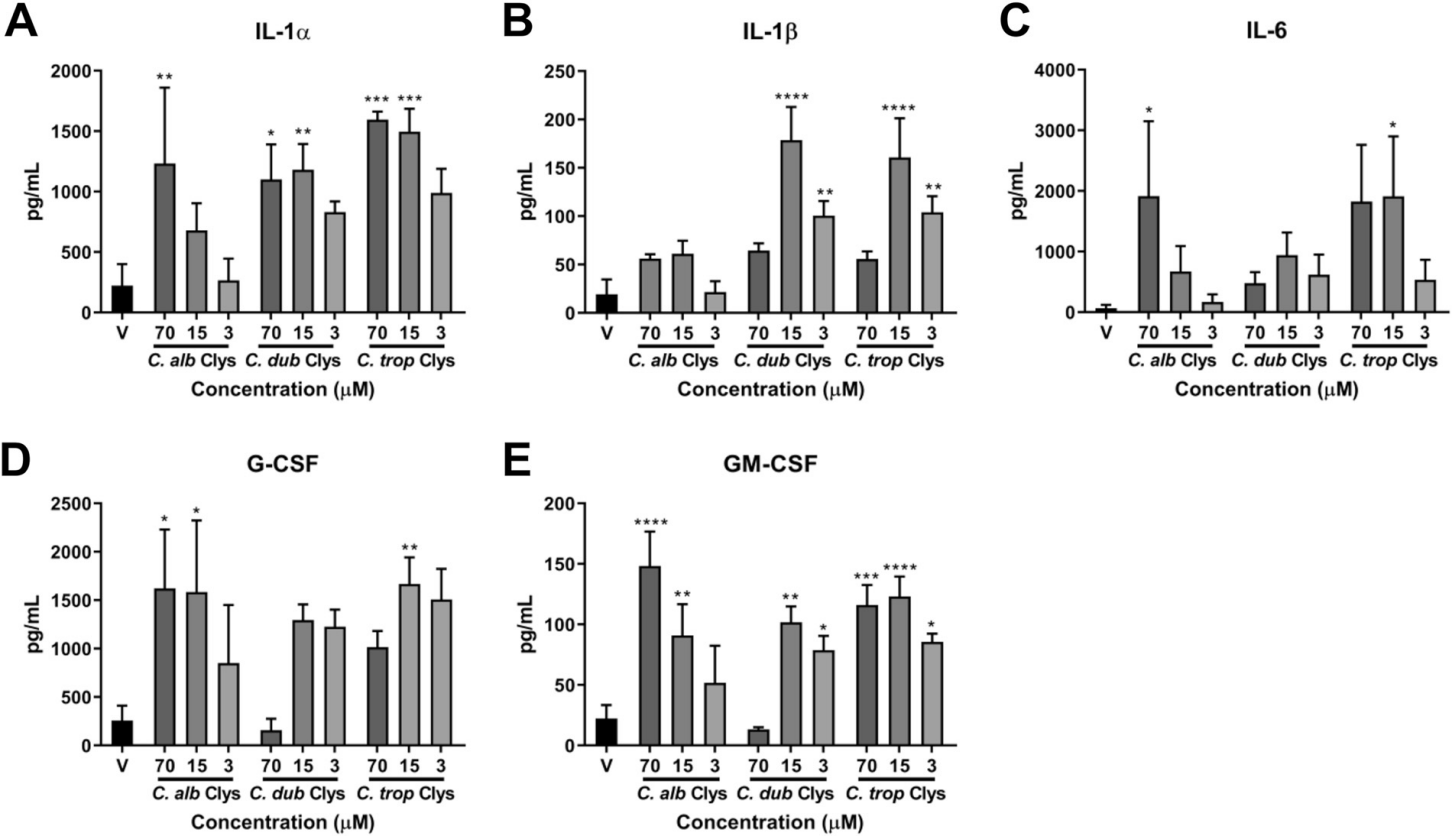
The candidalysins induce different levels of cytokine secretion. Quantification of cytokines (IL-1α, IL-1β, IL-6, G-CSF, and GM-CSF) secreted from epithelial cells treated with the candidalysins (70, 15, and 3 μM) of C. albicans, C. dubliniensis, and C. tropicalis is shown. Statistics are applied relative to vehicle-treated cells. Data are presented as means and SD from 3 biological repeats. For IL-1α, for C. dubliniensis candidalysin (15 μM) versus C. albicans candidalysin (15 μM), the *P* value was 0.0360 (*). For IL-1β, for C. dubliniensis and C. tropicalis candidalysins (15 μM) versus C. albicans candidalysin (15 μM), the *P* values were <0.0001 (****) and 0.0002 (***), respectively. For IL-1β, for C. tropicalis candidalysin (3 μM) versus C. albicans candidalysin (3 μM), the *P* value was 0.0021 (**). Statistical significance was calculated using one-way ANOVA with Tukey’s *post hoc* comparison test. ****, *P ≤ *0.0001; ***, *P ≤ *0.001; **, *P ≤ *0.01; *, *P ≤ *0.05.

Epithelial responses to C. albicans candidalysin culminate in the secretion of cytokines and chemokines required for the coordination of appropriate innate immune responses (3, 8, 12–14, 33). Therefore, we treated oral epithelial cells with all three candidalysins (70, 15, and 3 μM) for 24 h and quantified the secretion of interleukin-1α (IL-1α), IL-1β, IL-6, granulocyte colony-stimulating factor (G-CSF), and granulocyte-macrophage colony-stimulating factor (GM-CSF). All three candidalysins induced these cytokines in a dose-dependent manner; however, in keeping with our above-described findings, the C. dubliniensis and C. tropicalis candidalysins were more potent than C. albicans candidalysin at inducing IL-1α, IL-1β, and IL-6 at lower concentrations (15 and 3 μM) (Fig. 3A to E). C. dubliniensis candidalysin (15 μM) stimulated significantly more IL-1α secretion than did C. albicans candidalysin. C. dubliniensis and C. tropicalis candidalysins (15 μM) induced significantly more secretion of IL-1β, while C. tropicalis candidalysin (3 μM) induced significantly more secretion of IL-1β than with the same concentration of C. albicans candidalysin. Similar concentrations of G-CSF and GM-CSF were secreted in response to all three candidalysins, except for C. dubliniensis candidalysin at 70 μM, which failed to induce these two cytokines (Fig. 3D and E), presumably through excess toxicity. Collectively, these data demonstrate that the C. dubliniensis and C. tropicalis candidalysins are potent inducers of epithelial immune signaling mechanisms.

### C. dubliniensis and C. tropicalis candidalysins exhibit rapid real-time permeabilization of artificial lipid bilayers.

**FIG 4:**
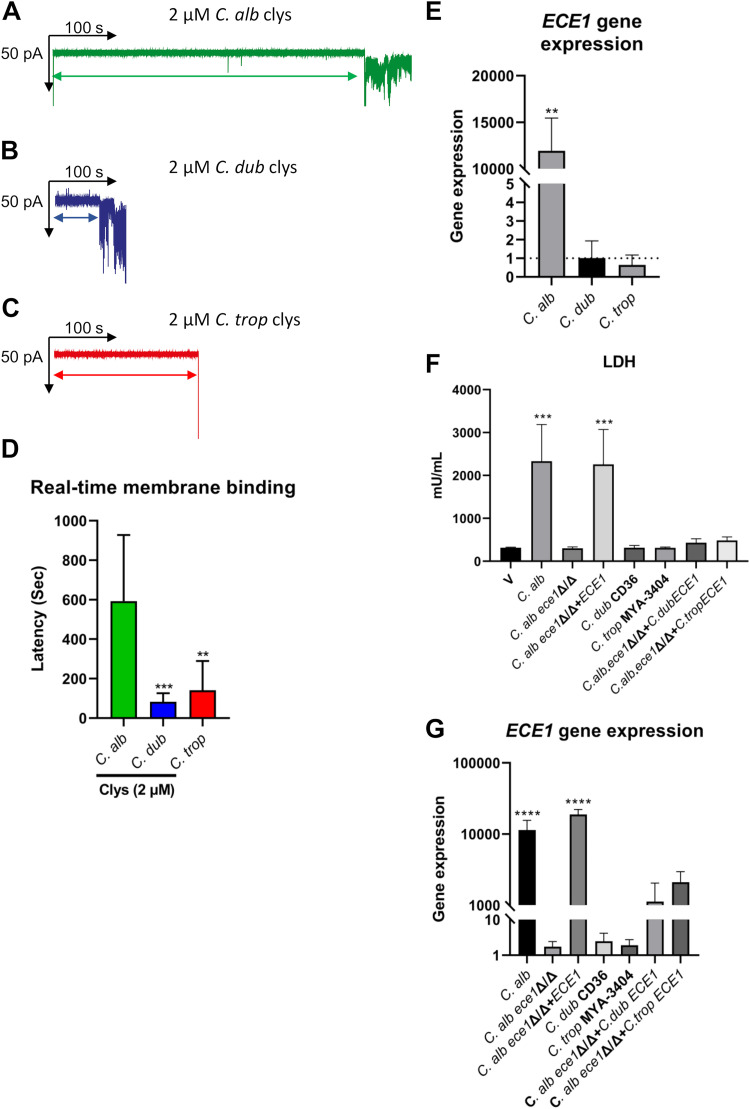
Candidalysins exhibit differences in real-time membrane permeabilization, and C. albicans, C. dubliniensis, and C. tropicalis fungi exhibit differences in *ECE1* gene expression. (A to C) Individual representative traces quantifying the dwell time (defined as the time that elapsed between candidalysin addition and bilayer permeabilization) for C. albicans (A), C. dubliniensis (B), and C. tropicalis (C) candidalysins at 2 μM. (D) Comparison of average dwell times for 2 μM candidalysins from C. albicans, C. dubliniensis, and C. tropicalis. Data are presented as means and SD from 10 biological repeats. Statistical significance was calculated using a two-tailed unpaired *t* test (versus C. albicans candidalysin). ***, *P ≤ *0.001; **, *P ≤ *0.01. (E) *ECE1* gene expression in C. albicans, C. dubliniensis, and C. tropicalis was quantified in the presence of epithelial cells by RT-qPCR after 24 h. Gene expression is presented relative to the 0-h samples cultured in YPD medium at 30°C in the absence of epithelial cells (dotted line). The fold change (2^ΔΔ^*^CT^*) was calculated for each strain using the threshold cycle method in comparison to *ACT1* as the reference gene. Data are the means and SD from 3 biological replicates. Statistical significance was calculated using a two-tailed unpaired *t* test (versus the time zero control). **, *P ≤ *0.01. (F) TR146 oral epithelial cells were infected with C. albicans expressing C. dubliniensis *ECE1* (*C.alb*.*ece1*Δ/Δ+*C.dubECE1*) and C. tropicalis *ECE1* (*C.alb*.*ece1*Δ/Δ+*C.tropECE1*) for 24 h, and levels of cell damage were assessed by an LDH assay. Statistics are applied relative to vehicle-treated cells. Data are the means and SD from 3 biological repeats. (G) *ECE1* gene expression in the *C.alb*.*ece1*Δ/Δ+*C.dubECE1* and *C.alb*.*ece1*Δ/Δ+*C.tropECE1* mutants was quantified in the presence of epithelial cells by RT-qPCR after 24 h. Gene expression is presented relative to the 0-h samples cultured in YPD medium at 30°C in the absence of epithelial cells. The fold change (2^ΔΔ^*^CT^*) was calculated for each strain using the threshold cycle method in comparison to *ACT1* as the reference gene. Data are the means and SD from 3 biological replicates. Statistical significance in panels F and G was calculated using one-way ANOVA with Tukey’s *post hoc* comparison test. ****, *P ≤ *0.0001; ***, *P ≤ *0.001.

To further investigate the observed difference in candidalysin potencies, we used Orbit 16 technology to quantify the real-time binding of all three candidalysins to artificial 1,2-diphytanoyl-*sn*-glycero-3-phosphocholine (DPhPC) planar lipid bilayers. Candidalysins (2, 4, 6, 8, and 10 μM) from C. albicans, C. dubliniensis, and C. tropicalis were applied to DPhPC planar lipid bilayers, and the dwell time (defined as the time that elapsed between candidalysin addition and bilayer permeabilization) was quantified (Fig. S1C). The candidalysins of C. dubliniensis and C. tropicalis were far more rapid (by a factor of 4 to 7) at permeabilizing a DPhPC bilayer than C. albicans candidalysin at a concentration of 2 μM (compare representative traces in Fig. 4A to C and averaged data in Fig. 4D). Collectively, these data demonstrate that candidalysins from C. dubliniensis and C. tropicalis permeabilize DPhPC membranes more rapidly than C. albicans candidalysin.

### C. albicans, C. dubliniensis, and C. tropicalis exhibit differences in *ECE1* gene expression.

Hyphal growth and maintenance are known to differ among C. albicans, C. dubliniensis, and C. tropicalis and between strains of each species (23, 25, 27). Since the expression of C. albicans *ECE1* (encoding candidalysin) is strongly induced during interactions with epithelial cells, we investigated the levels of *ECE1* gene expression between species during infection of oral epithelial cells. Oral epithelial cells were infected with C. albicans, C. dubliniensis, and C. tropicalis for 24 h, and *ECE1* gene expression was analyzed by reverse transcription-quantitative PCR (RT-qPCR). As expected, C. albicans expressed significant levels of *ECE1* (11,000-fold) in the presence of epithelial cells compared to the preculture. In contrast, no difference in *ECE1* gene expression was observed in C. dubliniensis or C. tropicalis in the presence of epithelial cells compared to the preculture (Fig. 4E). These data suggest that while the C. dubliniensis and C. tropicalis synthetic candidalysins are more potent than the C. albicans toxin when applied to oral epithelial cells, neither native candidalysin appears to be produced by representative strains of each species during infection of oral epithelial cells *in vitro*.

### Ectopic expression of C. dubliniensis and C. tropicalis *ECE1* in C. albicans.

A direct comparison of C. albicans, C. dubliniensis, and C. tropicalis pathogenicities is severely hampered by differences in hypha formation and elongation and *ECE1* gene expression. To mitigate these issues, we used CIp10 (34) to create plasmids where the *ECE1* genes of C. dubliniensis and C. tropicalis were placed under the control of the C. albicans SC5314 *ECE1* promoter. These constructs were introduced into a C. albicans *ece1*Δ/Δ null mutant (3) to enable comparisons of candidalysin activities without differences in hyphal growth and maintenance and gene expression. The integration of each construct into the C. albicans genome was verified by PCR analysis of genomic DNA (gDNA). All transformants were viable and displayed similar levels of filamentation when cultured on epithelial cells.

We infected oral epithelial cells with C. albicans harboring the *ECE1* gene of C. dubliniensis (*C.alb*.*ece1*Δ/Δ+*C.dubECE1*) or C. tropicalis (*C.alb*.*ece1*Δ/Δ+*C.tropECE1*) and assessed epithelial damage after 24 h by an LDH assay. C. albicans *ece1*Δ/Δ null and *ece1*Δ/Δ+*ECE1*, C. dubliniensis CD36, and C. tropicalis MYA-3404 were used as controls. All control species and strains behaved as expected, with C. albicans inducing cell damage in an *ECE1*-dependent manner (3) and C. dubliniensis and C. tropicalis exhibiting minimal levels of damage compared with C. albicans, as observed previously (23). Notably, only minimal levels of epithelial damage were caused by *C.alb*.*ece1*Δ/Δ+*C.dubECE1* and *C.alb*.*ece1*Δ/Δ+*C.tropECE1* (Fig. 4F). To determine if *ECE1* gene expression was impaired in these ectopic mutants, we infected epithelial cells with *C.alb*.*ece1*Δ/Δ+*C.dubECE1* and *C.alb*.*ece1*Δ/Δ+*C.tropECE1* for 24 h and quantified gene expression by RT-qPCR. As expected, the C. albicans parent strain and the C. albicans *ece1*Δ/Δ+*ECE1* mutant expressed significant levels of *ECE1* in the presence of epithelial cells, while wild-type (WT) C. dubliniensis CD36 and C. tropicalis MYA-3404 did not express *ECE1*. Surprisingly, while the *C.alb*.*ece1*Δ/Δ+*C.dubECE1* and *C.alb*.*ece1*Δ/Δ+*C.tropECE1* ectopic mutants also expressed *ECE1*, expression was much reduced compared with that of the C. albicans *ece1*Δ/Δ+*ECE1* control (Fig. 4G). These data demonstrate that the ectopic expression of C. dubliniensis and C. tropicalis *ECE1* in C. albicans fails to induce significant epithelial damage.

## DISCUSSION

Numerous pore-forming toxins are found in nature, including colicins, cytolysins, hemolysins, aerolysins, and cholesterol-dependent cytolysins of bacterial pathogens (reviewed in references 1 and 2); actinoporins of sea anemones (35); fungal killer toxins (36); and antimicrobial peptides (37). In 2016, we discovered candidalysin, the first cytolytic peptide toxin identified in a human fungal pathogen. Candidalysin is secreted by C. albicans and is critical for disease pathology during mucosal and systemic infections, the facilitation of fungal translocation across intestinal epithelial cells, and the amplification of immune responses (3, 8, 10, 14–16).

Although differences in the levels of *ECE1* gene expression between *Candida* species have been reported during vaginal infection *in vitro* and *in vivo* (24), it was unknown whether the C. dubliniensis and C. tropicalis *ECE1* genes also encode candidalysin peptide toxins and whether these are functional. We now identify the candidalysins as a new family of functionally active peptide toxins and the first family of peptide cytolysins in human-pathogenic fungi.

Ece1p and candidalysin orthologs were found in C. dubliniensis and C. tropicalis, which are pathogenic to humans and capable of forming hyphal filaments when cultured in the presence of epithelial cells (23). Notably, other pathogenic *Candida* species, including C. glabrata, C. parapsilosis, and the emerging pathogen C. auris, do not harbor Ece1p or candidalysin orthologs, which suggests that the damage potential and pathogenicity of these species are dependent on other fungal factors.

The candidalysins of C. albicans, C. dubliniensis, and C. tropicalis exhibit clear differences in potency. This was evidenced by (i) the greater LDH activity, more rapid calcium influx, and stronger c-Fos responses induced by the C. dubliniensis and C. tropicalis candidalysins than those induced by the C. albicans candidalysin; (ii) the greater release of the damage-associated cytokine IL-1α by the C. dubliniensis and C. tropicalis candidalysins but not the C. albicans candidalysin at lower concentrations (15 and 3 μM); and (iii) the significantly shorter time for C. dubliniensis and C. tropicalis candidalysins (2 μM) to permeabilize artificial DPhPC planar lipid bilayers than for C. albicans candidalysin (summarized in Table 1). The greater potency of C. dubliniensis and C. tropicalis candidalysins is an intrinsic property of their respective amino acid sequences since the DPhPC planar lipid bilayers used in the permeability assays did not contain heterologous protein (i.e., purified receptors).

**TABLE 1:** Physical properties and epithelial responses to candidalysins

Property	Strength of response to candidalysin from^*a*^:		
	C. albicans	C. dubliniensis	C. tropicalis
Amphipathicity	+++++	+++++	++++
α-Helicity	+++	++++	++++
Cellular damage	+++	+++++	+++++
Calcium influx	+++	+++++	+++++
MAPK signaling	+++	+++++	+++++
Cytokine secretion	+++	+++++	+++++
Membrane permeabilization	+++	+++++	++++

a +++, comparatively low potency; ++++, comparatively moderate potency; +++++, comparatively high potency.

Interestingly, while the C. dubliniensis and C. tropicalis candidalysins have more damaging and activating potential than C. albicans candidalysin, C. dubliniensis and C. tropicalis fungi are “less pathogenic” than C. albicans. This discrepancy in pathogenicity likely corresponds to the expression level and context of candidalysin processing, secretion, and delivery to the host membrane. We previously noted that in the presence of epithelial cells, hypha formation and maintenance in C. dubliniensis and C. tropicalis are poor compared to those in C. albicans SC5314 (23). Assuming that *ECE1* gene expression is associated with hypha formation in C. dubliniensis and C. tropicalis, similarly to C. albicans, it would be expected that *ECE1* expression (and, hence, candidalysin production) will also be far lower in these species. Indeed, clear differences in the levels of *ECE1* gene expression were observed in C. albicans, C. dubliniensis, and C. tropicalis when cultured in the presence of oral epithelial cells. Furthermore, similar analyses demonstrate that C. dubliniensis and C. tropicalis express far less *ECE1* than C. albicans when cultured on vaginal epithelial cells *in vitro*, and no *ECE1* expression was observed *in vivo* in a murine vaginitis infection model (24). Thus, the apparent disparity between the candidalysin potency and fungal pathogenicity of these three *Candida* species can be explained by differences in hypha formation, hypha maintenance, and levels of *ECE1* gene expression and candidalysin secretion during infection.

The C. albicans strain used in this study (SC5314) is more pathogenic and induces stronger damage than other clinical strains of C. albicans (38, 39). Notably, clinical C. albicans strains often exhibit impaired hyphal maintenance on epithelial cells compared with strain SC5314 (13, 39). Furthermore, *ECE1* gene expression in clinical strains only partially correlated with epithelial damage *in vitro*, independently of hypha formation (39). However, this discrepancy was recently clarified by a study that demonstrated that a combination of sustained hypha formation, *ECE1* expression, and candidalysin secretion into an invasion pocket was critical for sustained damage induced by invading *Candida* species during mucosal infection (40).

To mitigate differences in hypha formation and maintenance, *ECE1* gene expression, and invasion pocket formation among C. albicans, C. dubliniensis, and C. tropicalis, the *ECE1* genes of C. dubliniensis and C. tropicalis were placed under the control of the C. albicans *ECE1* promoter and introduced into a C. albicans *ece1*Δ/Δ mutant. The inability of the *C.alb*.*ece1*Δ/Δ+*C.dubECE1* and *C.alb*.*ece1*Δ/Δ+*C.tropECE1* ectopic mutants to cause epithelial damage was unexpected, but this was likely due to reduced *ECE1* gene expression levels resulting in insufficient accumulation of candidalysin in the invasion pocket for damage to occur. These observations suggest that there are additional levels of regulation of *ECE1* gene expression or transcript stability that prevent the creation of a strain that expresses similar levels of *ECE1* transcripts encoding candidalysins from different species.

The question remains as to the biological significance of a more potent toxin in a less pathogenic *Candida* species. One possibility is that these toxins are actively suppressed, via either reduced hypha formation or specific *ECE1* gene targeting, to prevent excess toxicity and immune induction. Another possibility is that the niches where the candidalysins of C. dubliniensis and C. tropicalis are strongly expressed for the benefit of the fungus have not yet been identified. Such questions can be addressed in future investigations.

In summary, this work identifies the candidalysins as a new family of fungal peptide toxins, which have similar properties but differing potencies.

## MATERIALS AND METHODS

### Candidalysin peptides.

The candidalysin peptides used in this study have the following amino acid sequences: SIIGIIMGILGNIPQVIQIIMSIVKAFKGNK for C. albicans candidalysin, SIIGILTAILNNVPQIINVITTIIKSITGNK for C. dubliniensis candidalysin, and ISFAGIVSSIINQLPSIIQIIGNIIKAGLVK for C. tropicalis candidalysin. All peptides were purchased from Peptide Protein Research Ltd. (UK). Each peptide was synthesized using standard 9-fluorenylmethoxy carbonyl (Fmoc) chemistry and purified by high-performance liquid chromatography (HPLC) to a minimum purity of 95%. Peptide purity and experimental molecular mass were further verified by liquid chromatography-tandem mass spectrometry (LC-MS/MS).

### Antibodies.

p-DUSP1/MKP1 (S359) and c-Fos rabbit monoclonal antibodies were purchased from Cell Signaling Technologies (catalog numbers 2857 and 2250, respectively). Actin (clone C4) mouse monoclonal antibody was purchased from Millipore (catalog number MAB1501). Peroxidase-conjugated AffiniPure goat anti-mouse and anti-rabbit IgG secondary antibodies were purchased from Jackson ImmunoResearch (catalog numbers 115-035-062 and 111-035-003, respectively).

### Oligonucleotides.

The following oligonucleotide primers (5′ to 3′) were used to quantify *ECE1* gene expression: forward (Fw) primer CTTTATCTTCTCAAGCTGC and reverse (Rev) primer CAACAACAGAATCAATATCTTC for C. albicans SC5314, Fw primer GCTGATCCTGTTGTTGCTGAACC and Rev primer ATGGCATATCAGCAATGACACCAG for C. dubliniensis CD36, Fw primer GATGCTGTCTTAGCTGGTTCTG and Rev primer CATCTCTCTTAACAAGGCCAGC for C. tropicalis MYA-3404, and Fw primer CCAGGTATTGCTGAACGTATGC and Rev primer GGACCAGATTCGTCGTATTCTTG for universal *ACT1*. The following oligonucleotide primers (5′ to 3′) were used to verify the genomic integration of CIp10 into the C. albicans genome: Fw primer CGCCAAAGAGTTTCCCCTATTATC (RPF-2) and Rev primer CACAACAGAGCTTCTAAC (ECE-check1) for the 5′ integration site and Fw primer GGAGTTGGATTAGATGATAAAGGTGATGG (URA-F2) and Rev primer GAGCAGTGTACACACACACATCTTG (RPF-1) for the 3′ integration site.

### Plasmid construction.

A CIp10 plasmid (34) containing an MluI/SalI insert comprising 3,128 bp of 5′ intergenic sequence, the C. albicans *ECE1* gene, and 373 bp of 3′ intergenic sequence (3) was modified to express the *ECE1* genes of C. dubliniensis and C. tropicalis. Plasmid inserts were synthesized and cloned into recipient CIp10 by GeneArt (Thermo Fisher). Briefly, the C. albicans *ECE1* gene was excised from the CIp10 plasmid described above and replaced with C. dubliniensis *ECE1* and C. tropicalis *ECE1* to yield CIp10 constructs containing *ECE1* from C. albicans, C. dubliniensis, and C. tropicalis with identical 5′- and 3′-flanking intergenic sequences. The sequence of each insert was verified by DNA sequencing. Constructs were linearized by digestion with StuI prior to transformation.

### Transformation of C. albicans and extraction of genomic DNA.

A uridine-auxotrophic *ece1*-null mutant [*ece1*Δ/Δ (*ura^−^*)] was transformed with 15 μg of linearized plasmid DNA using a lithium acetate method modified from a method described previously (41). Transformants were selected on minimal (SD) agar medium and restreaked onto fresh SD agar to ensure stability, and genomic DNA was extracted using phenol-chloroform-isoamyl alcohol and glass bead lysis. The successful integration of each construct into the C. albicans genome (at the RPS1 locus) was confirmed by PCR amplification across the 5′ and 3′ integration sites.

### Mammalian cell culture.

All experiments were performed using the TR146 human oral epithelial cell line (42) purchased from the European Collection of Authenticated Cell Cultures. Cultures were verified to be mycoplasma-free by PCR. Cells were cultured in a Dulbecco modified Eagle medium (DMEM)–F-12 medium nutrient mixture (1:1) plus l-glutamine (Life Technologies) supplemented with 15% (vol/vol) heat-inactivated fetal bovine serum (Life Technologies) and 1% (vol/vol) penicillin-streptomycin (Sigma) at 37°C with 5% CO_2_.

### Fungal culture, infection of epithelial cells, and induction of hyphal growth.

C. albicans SC5314, C. dubliniensis CD36, and C. tropicalis MYA-3404 were cultured in yeast extract-peptone-dextrose (YPD) liquid medium overnight at 30°C in a shaking incubator at 180 rpm. Fungi were washed twice in phosphate-buffered saline (PBS), added to epithelial cells at a multiplicity of infection (MOI) of 0.01, and incubated at 37°C with 5% CO_2_ in a humidified incubator for 24 h. Induction of hyphal growth was performed in the presence of TR146 oral epithelial cells (MOI of 0.01) for 24 h.

### Circular dichroism spectroscopy.

Candidalysin peptides were reconstituted in sterile water (10 mg/mL stock concentration) and further diluted to 0.2 mg/mL in buffer (10 mM Tris [pH 7.0], 50 mM NaCl). Circular dichroism was performed using a Chirascan spectrometer (Applied Photophysics) at 20°C, at wavelengths of 190 to 260 nm with intervals of a 0.5 bandwidth, 2 s, and 2 repeats for each acquisition point. CD spectra were acquired using a 1-mm-path-length quartz cuvette (100-QS). All acquired spectra were averaged and corrected for background and buffer contributions, and the net spectra were smoothed with a Savitzky-Golay filter (window 3).

### Treatment of epithelial cells with candidalysin peptides.

Prior to infection, confluent oral epithelial cells were serum starved overnight, and all experiments were carried out in serum-free DMEM–F-12 medium. Epithelial cells were treated with candidalysin peptides at the indicated concentrations for maxima of 24 h (damage and cytokine assays), 3 h (calcium), and 2 h (Western blotting). Treated cells were cultured at 37°C with 5% CO_2_.

### Epithelial cell damage assay.

Damage to oral epithelial cells was quantified using a Cytox 96 nonradioactive cytotoxicity assay kit (Promega) according to the manufacturer’s instructions. Recombinant porcine lactate dehydrogenase (Sigma) was used to create a standard curve.

### Quantification of calcium influx.

Epithelial cells were seeded into opaque, clear-bottomed, 96-well plates (Greiner) at a density of 5 × 10^5^ cells/mL; cultured to confluence overnight; and serum starved for 24 h. A solution containing 2.5 μM Fura-2 AM (Thermo Scientific) and 500 μM probenecid (Sigma) was prepared in a saline solution (140 mM NaCl, 5 mM KCl, 1 mM MgCl_2_, 2 mM CaCl_2_, 10 mM glucose, 10 mM HEPES [pH 7.4]). Serum-free medium was removed and replaced with 50 μL of a Fura-2 AM–probenecid-containing saline solution, and the mixture was incubated at 37°C with 5% CO_2_ in the dark for 60 min. Following incubation, the Fura-2 AM–probenecid solution was removed and replaced with 50 μL of a saline solution. The plate was read on a FlexStation 3 multimode microplate reader (Molecular Devices). Samples were excited at 240/280 nm, and fluorescence was detected at 520 nm. For 30-min time course recordings (Fig. 2C), readings were taken every 15 s. For 180-min time course recordings (see Fig. S1 in the supplemental material), readings were taken every 60 s for the first 30 min and then every 5 min for 2.5 h. Results were expressed as a ratio between 340 and 380 nm.

### Protein extraction from TR146 oral epithelial cells.

Tissue culture plates were placed on ice, and cells were washed with 1 mL of ice-cold PBS and then lysed with 120 μL of a modified radioimmunoprecipitation assay (RIPA) buffer (50 mM Tris-HCl [pH 7.4], 150 mM NaCl, 1 mM EDTA, 1% Triton X-100, 1% sodium deoxycholate, 0.1% SDS) supplemented with 1× protease inhibitors (Thermo Scientific) and 1× phosphatase inhibitors (Sigma). A sterile cell scraper was used to detach cells from the surface of the plate. After scraping, crude cell extracts were collected, transferred to microcentrifuge tubes, and incubated on ice for 30 min. Following incubation, extracts were clarified by centrifugation in a benchtop microcentrifuge at 16,200 × *g* at 4°C for 10 min. Clarified extracts were collected, and the protein concentration was estimated using a bicinchoninic acid assay (Thermo Scientific) according to the manufacturer’s instructions.

### SDS-PAGE and Western blotting.

Proteins were resolved by electrophoresis on 12% SDS-PAGE gels using a mini-Protean Tetra cell system (Bio-Rad). Electrophoresed proteins were transferred to a nitrocellulose membrane (Bio-Rad) using a mini-Transblot electrophoretic transfer cell (Bio-Rad). Membranes were blocked in 1× Tris-buffered saline (TBS; Severn Biotech) containing 0.001% (vol/vol) Tween 20 (Acros Organics) and 5% (wt/vol) fat-free milk powder (Sainsbury’s). c-Fos, p-DUSP1/MKP1, and α-actin primary antibodies were diluted (1:3,000, 1:1,000, and 1:10,000, respectively) in TBS-Tween and 5% milk, and membranes were incubated overnight at 4°C with gentle shaking (c-Fos and p-DUSP1/MKP1) or for 1 h at room temperature with gentle shaking (α-actin). Following incubation, membranes were washed with 1× TBS containing 0.001% (vol/vol) Tween 20, diluted (1:10,000) horseradish peroxidase (HRP)-conjugated secondary antibody was added, and membranes were incubated for 1 h at room temperature. Membranes were washed as described above and exposed to the Immobilon Western chemiluminescent HRP substrate (Millipore) prior to visualization by exposure to film (GE Healthcare). α-Actin was used as a loading control.

### Quantification of cytokine release from TR146 oral epithelial cells.

Exhausted cell culture medium was collected, and the concentration of cytokines was determined using magnetic microparticles (R&D Systems) specific for human IL-1α, IL-1β, IL-6, G-CSF, and GM-CSF using a magnetic Luminex performance assay (Bio-Techne) and the Bio-Plex 200 system (Bio-Rad) according to the manufacturers’ instructions. Data were analyzed using Bioplex Manager 6.1 software.

### Quantification of bilayer permeabilization.

Current measurements were performed using multiple planar lipid bilayers and an Orbit 16 system (Nanion). The horizontal bilayers were formed over 16-channel multielectrode-cavity-array (MECA) chips (Ionera) using 1,2-diphytanoyl-*sn*-glycero-3-phosphocholine (DPhPC) lipids (Avanti Polar Lipids) dissolved in octane (25 mg mL^−1^). Both *cis* (grounded) and *trans* cavities above and below the bilayers were filled with an electrolyte solution containing 0.1 M KCl and 20 mM HEPES (pH 7.4). Candidalysin peptides dissolved in water were added to the *cis* side of bilayers at final concentrations of 2, 4, 6, 8, and 10 μM. A constant voltage of −50 mV was applied, and current changes were monitored for 10 min at room temperature. Current traces were acquired at a sampling frequency of 10 kHz using Element Data Recorder software (EDR 3.8.3). Current analysis was performed using Clampfit 10.3 (Molecular Devices). Plots were generated using GraphPad software.

### RNA extraction.

Epithelial cells were infected with *Candida* species for 24 h (MOI = 0.01). Exhausted culture medium was collected, and nonadherent fungi were pelleted by centrifugation. Adherent fungi were rinsed with ice-cold PBS, loosened with a cell scraper, and added to the pelleted sample. Fungi were washed with 1 mL of ice-cold PBS, resuspended in a final volume of 500 μL, added to a cryovial containing an ∼1/3 volume of acid-washed glass beads (0.5 mm; Thistle Scientific), and bead beaten four times at 4.5 m/s^2^ for 30 s using a FastPrep-24 system (MP Bio). The lysates were removed, and RNA was extracted using a MasterPure yeast RNA purification kit (Lucigen) according to the manufacturer’s instructions. The removal of contaminating gDNA was performed according to kit instructions. For the 0-h control samples, RNA was extracted from 500 μL of each YPD fungal culture in the absence of epithelial cells.

### Quantification of *ECE1* gene expression.

cDNA was synthesized using a QuantiTect reverse transcription kit (Qiagen) and 600 ng of the RNA template. cDNA samples were then used for qPCR using Hot FIREPol EvaGreen qPCR supermix (Solis BioDyne). Primers (universal forward and reverse primers for *ACT1* and species-specific forward and reverse primers for *ECE1*) were used at a final concentration of 200 nM. qPCR amplifications were performed using a Rotor-gene system (Corbett). *ECE1* gene expression was calculated individually for each strain using the threshold cycle (ΔΔ*C_T_*) method to calculate fold changes (2^ΔΔ^*^CT^*) and using *ACT1* as the reference gene. Data were represented by comparing fold changes in expression after setting controls (YPD at time zero) to a value of 1.

### Statistical analysis of data.

Data sets of three or more were analyzed by one-way analysis of variance (ANOVA) with Tukey’s *post hoc* comparison test using GraphPad Prism 9 software. Pairwise comparisons of data were analyzed using a two-tailed unpaired *t* test using GraphPad Prism 9 software. In all cases, a *P* value of *≤*0.05 was taken to be significant.
