# Supplementary figures and images for: Candidalysins Are a New Family of Cytolytic Fungal Peptide Toxins
Source: mBio. 2022 Jan 25;13(1):e03510-21. doi: 10.1128/mbio.03510-21 (PMC8787473; doi:10.1128/mbio.03510-21)

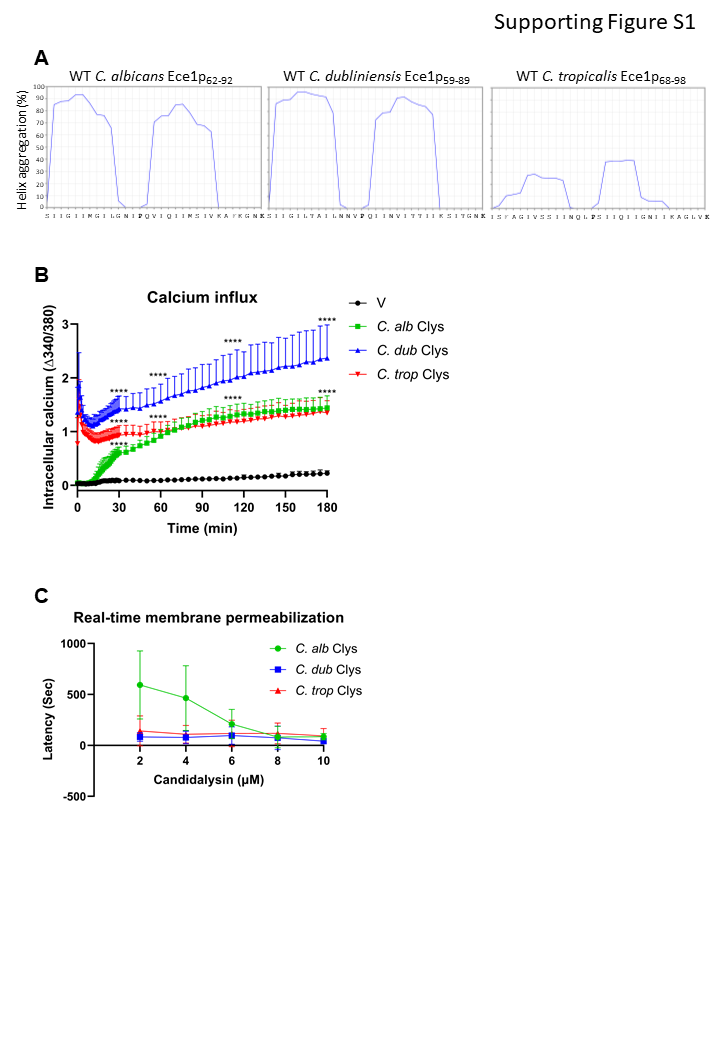

Supplement: FIG S1 [file mbio.03510-21-sf001.tif]
